# Effects of Dietary Protein on Thyroid Axis Activity

**DOI:** 10.3390/nu10010005

**Published:** 2017-12-22

**Authors:** Ewelina Pałkowska-Goździk, Katarzyna Lachowicz, Danuta Rosołowska-Huszcz

**Affiliations:** Department of Dietetics, Faculty of Human Nutrition and Consumer Sciences, Warsaw University of Life Sciences WULS-SGGW, 159c Nowoursynowska Str., 02-776 Warsaw, Poland; katarzyna_lachowicz@sggw.pl (K.L.); danuta_rosolowska_huszcz@sggw.pl (D.R.-H.)

**Keywords:** thyroid hormones, thyroxine, triiodothyronine, thyrotropin, dietary proteins, diet, amino acids

## Abstract

Thyroid hormones (TH) are essential for the normal development and function of every vertebrate. The hypothalamic-pituitary-thyroid (HPT) axis is regulated to maintain euthyroid status. One of the most influential environmental factors that determines HPT axis activity is nutrition. Both food availability and substrate diversity affect thyroid hormone economy. The present paper aims to summarize literature data concerning the influence of the amount and the type of protein on thyroid axis activity. This review sheds light on the contribution of a low-protein diet or insufficient intake of essential amino acids to TH abnormalities. We believe that the knowledge of these dependencies could improve the results of nutritional interventions in thyroid axis disorders and enhance the efficiency of animal breeding.

## 1. Introduction

Thyroid hormones (TH) play a crucial role in the differentiation, growth, development and function of nearly all tissues. They have long been recognized as a key regulator of oxygen consumption and the basal metabolic rate [1]. These effects have been attributed to the direct actions of TH on the heart and metabolically active tissues, including liver, white and brown adipose tissue and skeletal muscle [2]. Thyroid hormones are also involved in the central regulation of energy balance at the level of the hypothalamus [3].

Serum thyroid hormone levels are tightly regulated by a negative feedback mechanism joining the hypothalamus, pituitary and thyroid glands in a precisely functioning axis (HPT) [4]. The central regulation of the HPT axis by hypophysiotropic thyrotropin-releasing hormone (TRH) neurons in the hypothalamic paraventricular nucleus (PVN) is of key importance for adaptive changes in thyroid axis activity in response to external and internal stimuli [5].

The main hormone secreted by the thyroid is 3,3′,5,5′ tetraiodothyronine (thyroxine, T4), which, in target tissues, undergoes 5′ deiodination to metabolically active 3,3′,5 triiodothyronine (T3) or 5 deiodination to inactive 3,3′,5′ reverse triiodothyronine (rT3). Both 5 and 5′ deiodination are catalysed by deiodinase type 1 (D1); deiodinase type 2 (D2) catalyses 5′ deiodination and deiodinase type 3 (D3) is engaged in 5 deiodination only.

Besides the negative feedback regulation of TRH synthesis and secretion by thyroid hormone, hypophysiotropic TRH neurons in the PVN receive afferents from catecholaminergic and hypothalamic arcuate nucleus (ARC) neurons [6]. One group of ARC neurons expresses anorexigenic α-melanocyte-stimulating hormone (α-MSH) and cocaine- and amphetamine-regulated transcript (CART) neuropeptides, which stimulate TRH secretion; the second group synthesize orexigenic neuropeptides Y (NPY) and Agouti-related peptide (AgRP), which in turn inhibits TRH release. Both of these neuron groups are regulated by metabolic and hormonal signals, including leptin, insulin, peptide YY (PYY) and ghrelin [7]. The integration of those inputs provides an exact set point for thyroid axis activity in response to nutritional alterations.

The food—both its quantity and quality—is one of the most influential environmental factors that determine HPT axis activity [8]. Food restriction down regulates HPT axis activity, which is assumed to be an energy-saving mechanism [9]. Starvation decreases TRH and TSH-β gene expression, circulating thyroid hormone levels in rodents and humans [9]. It was shown that the observed reduced T3 concentration results from decreased thyroidal secretion of T4 [10] or is, at least partially, induced by increased D3 activity in the liver, kidney and muscles of starved rats [11]. Using double knockout mice that lack both the melanocortin 4 receptor (MC4R) and NPY it was demonstrated that NPY plays a major role in the fasting-induced suppression of the HPT axis at the central level. Further, both MC4R and NPY were shown to be responsible for decreasing plasma hormone levels during food deprivation by TH sulphation or glucuronidation in the liver [12]. Another possible mechanism of plasma T3 decline could be associated with FOXO-1-mediated D2 inhibition, reversible by the activation of the PI3K-mTORC2-Akt pathway [8]. Not only does the amount of food supply affect thyroid hormone economy but also HPT axis activity responds to diet composition. Protein content in diet changes depending on various conditions. It could be increased or decreased because of health conditions or specific targets of dietary interventions. Dietary protein can markedly influence food intake by affecting physiological and neurobiological homeostatic regulatory mechanisms [13]. As thyroid activity is essential for proper development, metabolic regulations and for maintenance of energy balance, we aimed to review literature data concerning the effects of quantity and type of protein consumed on thyroid axis activity. A better understanding of this issue could also improve either the results of nutritional interventions for preventing/treating thyroid axis disorders or the efficiency of animal breeding.

The search for potentially relevant studies was performed using electronic databases PubMed/MEDLINE and EBSCO. The search terms included the combination of terms for thyroid (thyroid hormone, thyroid-stimulating hormone, thyroid axis activity or hypothalamic-pituitary-thyroid axis) and dietary protein (protein in the diet, protein source). The reference lists of articles included were also reviewed in an attempt to detect any other potentially eligible studies. Only articles in English and published from 1975 up to 31 May 2017 were retained for further assessment of eligibility. After removing of duplicate studies from the different search engines, independent screening of the title and abstract of the remaining articles was undertaken. Selected papers were then read in full to confirm the eligibility. 

## 2. Effect of Protein Quantity on HPT Axis Activity

The majority of available data concerning the effect of reduced diet protein content on thyroid axis activity.

The diminution of protein content in diets alters HPT axis activity at both central and peripheral levels with the effect similar to that caused by starvation [14]. Compared to rats on a control diet, rats fed a protein-free diet showed a significant decline in hypothalamic TRH, pituitary TSH-β transcript and plasma T3 concentrations [14]. Those changes were most likely evoked by an increase in the NPY level. It was shown that in rats fed a protein-restricted diet, hypothalamic NPY gene expression approached the level of energy-restricted animals and was significantly higher than in animals receiving control, or high protein, low-carbohydrate and low-fat diets. Furthermore, animals fed a low-protein diet were hyperphagic with increased adiposity compared to others [15]. Similar relationship between diet protein content and NPY level was demonstrated in the offspring of dams fed a low-protein diet (8% protein) during pregnancy and lactation and those receiving a normal protein diet (20% protein) [16]. The maternal poor-protein diet, as compared to a normal-protein diet, induced metabolic alterations, more pronounced in females than in male offspring, including elevated circulating NPY levels and up-regulation of NPY signalling in the visceral adipose tissue. This impaired metabolic phenotype was followed by a lower birth weight, increased abdominal adiposity and glucose intolerance after weaning onto a high-fat diet (20% protein, 35% carbohydrate, 45% fat), which confirms that protein malnutrition affects the metabolic and hormonal profiles in mice progeny [16]. Analogous conclusions were drawn by Zambrano et al. [16]. Wistar rats exposed to protein restriction (10% casein in the mother’s diet) during the prenatal and/or early postnatal period had lower body weight, decreased leptin and insulin sensitivity followed by higher cholesterol and triglycerides concentrations than those whose mothers were on a control diet (20% casein) [17]. Overall, protein deprivation throughout pre- and early postnatal periods increases the risk of developing the metabolic syndrome in later life [18]. Additionally, the exposure to a protein-restricted diet throughout those critical life stages permanently changes thyroid function in the progeny [19,20,21]. Protein malnutrition during gestation affects the structure of the thyroid in foetal and neonatal pups, leading to a delay in follicle organization and to a reduction in thyroid volume, which result from the reduced follicle number and thyrocyte size [19]. Compared with the offspring of control-fed dams, the new-borns of protein-restricted dams had lower T3 [22] and T4 as well as higher rT3 serum levels [23]. In lactating dams, protein restriction was associated with lower body weight but with increased serum free T3 (fT3) concentration, radioiodine mammary gland uptake and milk content as compared to controls, which was considered to be adaptation mechanisms designed to protect from neonatal hypothyroidism [24].

In the adult offspring of mothers fed a diet containing 8% protein, significantly higher thyroid radioiodine uptake, T3 and T4 concentrations were observed compared to the controls [21]. Maternal malnutrition programs higher hepatic D1 [25] and muscle D1 activity [20], which suggest hyperthyroid status in the progeny. The higher pituitary D2 activity in the offspring of protein-restricted dams could explain a decreased TSH response to in vitro TRH stimulation by increased local T3 generation [20]. The recorded increase in the serum fT3 level in calves exposed to maternal protein-restricted feed (70% of the recommended daily crude protein intake) in the second trimester of gestation was explained by increased prenatal glucocorticoid–stimulated conversion of T4 to T3, without a concomitant increase in TH-binding protein levels [26]. The fT3 contribution to the “catch-up growth” effect of these hyperthyroid low-birth-weight calves was also suggested since fT3 was positively correlated with the average daily gain rate [26]. In rats born to the dams exposed to low-protein diets during gestation, the reduced birth weight was followed by higher feed efficiency, raised core body temperature, higher resting and basal metabolic rates in the fed and fasted states compared to the controls [27]. 

In the hyperthyroid adult offspring of dams fed a protein-deficient diet (8% protein), the serum leptin level remained unchanged as compared to the controls despite a lower body weight resulting from a significant reduction in depots of visceral and subcutaneous adipose tissue [28]. The higher leptin to visceral fat ratio in the offspring of protein-malnourished mothers was accompanied by lower hypothalamic signal transducer and activator of transcription 3 (STAT3), higher pituitary leptin receptor (Ob-R) and lower thyroid Janus tyrosine kinase 2 (JAK2) contents compared to the adult progeny of dams fed a control diet (23% protein) [28]. Hyperthyroidism together with adipocyte hypotrophy and alteration in leptin signalling pathway in the regulation of HPT axis activity present in the adult protein-restricted offspring reinforce the concept of neonatal programming of thyroid hormone metabolism at peripheral and central levels by maternal nutrition [28]. 

Further, a low level of protein in the diet combined with iodine excess evoked a more extensive cellular damage of the thyroid gland than iodine overdose alone [29]. In Wistar rats fed a 6-month protein-restricted diet containing 50- and 100-fold excess iodine, a significant increase in the volume of thyroid follicles, the number of apoptotic cells and the flattening of epithelial cells were shown. Those abnormalities in thyroid histology were accompanied by lower T4, T3, fT4 and fT3 serum concentrations than observed in groups receiving the same amount of iodine but without protein restriction [29]. 

The recommended daily intake of a protein associated with an energy restriction induced a decrease in T3 serum concentration in normal-weight healthy men and women compared to those on a typical western diet. In contrast, there were no differences in TSH, T4 and fT4 concentrations between the groups [30]. In the study conducted on rats, the influence of a normal-protein and calorie-restricted diet on thyroid hormone levels has been shown to depend on age. In 17-week old rats, a decrease in TSH level, both total and free fraction of T3 and T4 was observed, whereas in 44-week old animals only T4, free T4 (fT4) and fT3 serum levels were affected by energy restriction [31]. Both caloric restriction connected with lower carbohydrate content as well as the higher protein to carbohydrate ratio could be the cause of these effects. In the early studies, low-protein high-carbohydrate diets were shown to increase T3 serum concentrations [32,33] and decrease fT3, T4 and TSH concentrations [34]. According to Lunn and Austin [32], T3 increase with a concomitant protein decline and an increase in energy/carbohydrate intake results from unbalanced consumption of these macronutrients since T3 did not increase if low-protein intake was accompanied by low-carbohydrate consumption. The decrease in fT3 concentration on low-protein high-carbohydrate diets was accompanied by an increase in T3 binding affinity by thyroid hormone transport proteins and even a direct correlation between fT3 level and protein intake was recorded [33]. 

A low-protein diet affects thyroxine-binding globulin (TBG) and transthyretin (TTR) concentrations in a different manner [35]. In adult rats fed low-protein diets (6% protein), re-expression of TBG disappearing during maturation resulted in its increased plasma level [35]. Contrary to this, a low-protein diet evoked a decrease in TTR plasma concentration [35] and expression in the liver [36].

On the other hand, in obese middle-aged women, exposure to a very low-energy but high-protein diet (daily dose of 70 g protein/420 kcal) caused a significant weight loss followed by a reduction in T3 concentration and a decrease in the resting metabolic rate. The decline in both parameters occurred permanent and even after a re-alimentation period, these parameters did not reach pre-diet values. Surprisingly, T4 and fT4 levels remained unchanged [37].

The status of plasma TH was also shown do be dependent on the feeding regime, both feeding frequency and feeding level [38,39]. More frequent meals enhance the efficiency of energy and protein utilization influencing postprandial hormone responses. Plasma T3 and T4 concentrations in heavy veal calves fed four times daily were higher than in those given normal-protein meal once a day [39]. A positive correlation between TH level and feeding level was also documented [38]. In calves exposed to a higher nutrient load (2.5 × metabolizable energy requirements for maintenance, MEm) that was distributed equally across multiple meals, higher T3 and T4 levels were observed compared to the low feeding level (1.5 × MEm) [39]. 

The details of cited studies are summarized in Table 1 in order of appearance in the text. 

## 3. Effect of Protein Source on HPT Axis Activity

The source of consumed protein has a significant impact on the thyroid hormone status. It was shown that some of the sulphur and aromatic amino acids derived from acidic hydrolysis of casein were able to inhibit in vitro the iodide oxidation reaction catalysed by thyroid peroxidase (TPO) [40]. Although cysteine and methionine or tyrosine and tryptophan were demonstrated to impair TPO iodination activity, their inhibitory potential was reversible with higher iodide concentrations [40]. There is also evidence that the excitatory amino acids (EAA) such as glutamate and aspartate are able to modify hormone secretion from the pituitary-thyroid axis [41]. Intraperitoneal administration of glutamate (l-Glu, 20 and 25 mg/kg) and *N*-methyl-d-aspartate (NMDA, 25 mg/kg) increased serum T4, T3 and TSH concentrations in freely moving adult male rats [42]. 

The long-term insufficient intake of dietary essential amino acids alters thyroid axis activity [43,44]. The deficiencies of these nutrients vary in their individual effects on thyroid hormone plasma concentrations. Carew et al. [43] described an increase in plasma T3 concentrations in broiler chicken receiving arginine-, lysine- or methionine-deficient diets (60% of recommended dietary allowances compared to pair-fed controls. The same outcome accompanied isoleucine deficiency (75% of recommended feed content). In turn, the threonine reduction had no effect on plasma T3 levels. Contrary to this, T4 plasma level was reduced only by the lysine restriction. Possible explanations for these results could be an increased thyroid gland hormone secretion, altered T4 to T3 conversion or changes in hormone clearance [43]. In another study, the influence of protein type was demonstrated to be significant only after a 50% diminution of the recommended protein dose [44]. At the adequate protein intake, there were no significant differences in total and free T3 and T4 concentrations between pigs receiving casein reinforced with limiting amino acids (cysteine, methionine, threonine and tryptophan—CAS+) and pigs fed a diet containing soy protein isolate without supplementation (SPI-). The 50% reduction of dietary protein content evoked an increase in T4 plasma levels in both dietary groups but T3 and fT3 concentrations were higher in pigs fed a CAS+ diet than in a SPI- diet given. Different endocrine responses observed in animals on protein-restricted and adequate feeding might result from decreased peripheral 5′-monodeiodination in rats given dietary protein of lower biological value. Moreover, independently of the amount of protein consumed, lower protein energy retention (PER) in SPI- was compensated by elevated fat energy retention (FER) compared to CAS+ [44].

In three groups of rats fed isonitrogenous (10% energy from protein daily) diets based on either casein with methionine, or on soy protein isolate and soy protein isolate supplemented with methionine, no differences in T3 and fT3 concentrations were observed but both T4 and fT4 concentrations as well as the levels of cholesterol and triglycerides, were seen to be lower in soy protein-fed rats than in the casein-fed group [45]. The diet containing 20% alcohol-washed soy protein isolate (SPI) upregulated the expression of the thyroid hormone receptor β-1 (TRβ-1) in the liver without altering thyroid hormone concentrations in rats. Therefore, via modulation of hepatic TRβ-1 protein content, SPI- might regulate the expression of genes involved in lipid metabolism, thereby exerting their lipid-lowering action [46]. 

The effects exerted by soy proteins may vary depending on their composition. Potter et al. [47] used diets containing an equal amount of protein from isolated soy protein (ISP), soy protein concentrate (SPC), or casein to assess their influence on thyroid hormone serum concentrations in Syrian hamsters. In animals receiving SPC serum, T4, fT4 and T3 levels were significantly lower than in ISP- or casein-fed hamsters. Given that the average protein content of isolated soy protein amounts to 90% and of soy protein concentrate to 70%, the number of other potentially biologically active compounds including isoflavones, saponins, phytic acid and trypsin inhibitors is higher in SPC than in ISP, resulting in differential hormonal responses [48].

Isoflavones, the main component of soybeans, influence thyroid axis activity [49,50]. Data from both in vitro and in vivo studies indicated that genistein, one of the soy isoflavones, is a potent inhibitor of TPO activity [50,51,52]. It was found that genistein and daidzein are able to suppress TPO-catalysed iodination and coupling in a dose-dependent manner. However, this effect occurred to be reversible when the incubation mixture was supplemented with iodide [53]. Comparable observations were made by Chang and Doerge [52]. Although TPO activity was inversely related to genistein doses in the diets, T3, T4 and TSH concentrations, thyroid weights and histology showed no differences between rats fed a standard soy-based and a soy-free diet [52]. Similarly, the profiles of thyroid hormones in rats exposed to genistein at two doses (1 and 5 mg/kg) were no significantly different from those set as controls [54]. 

In contrast, in cats submitted to a soy-based diet for 3 months, significantly higher T4 and fT4 but unchanged T3 levels were observed compared to animals on a soy-free diet [55]. Therefore, it was assumed that a short-term administration of a diet fortified with soy isoflavones exerts an inhibitory effect on 5′-iodothyronine deiodinase or accelerates T3 clearance [55]. In turn, a higher amount of free T4 might result from reduced TBG plasma levels after the soy isoflavones supplementation [56]. Further, in orchidectomized middle-aged rats, genistein or daidzein injected subcutaneously (10 mg/kg) induced follicular changes in the thyroid tissue, including the increased volume of Tg-immunopositive follicular epithelium and reduced volume of luminal colloid, higher TSH accompanied by lower T3 and T4 plasma levels as compared to the controls [57]. In adult female monkeys after chemical menopause, a long-term (34 months) administration of soy protein caused T3 increase without changes in TSH and T4 concentrations [58]. Ovariectomized sheep exposed to isoflavone-rich diet (81–95 mg phytoestrogens/kg from red clover silage) revealed higher T3 and T4 serum concentrations than hay-fed animals. The interactions between isoflavones and thyroidal oestrogen receptors were supposed to be involved in this stimulatory effect [59]. Persky et al. [60] showed that the 90 mg daily dose of isoflavones administered for 6 months was able to increase T3 and TSH concentrations in hypercholesterolemic postmenopausal women. However, there were no significant differences in the thyroid hormone status between postmenopausal women subjected to a diet supplemented with 90 mg (aglycone weight) of isoflavones per day and placebo group after 90 and 180 days of treatment [61]. Similar results were obtained by Mittal et al. [60] who showed that soy isoflavones (75 mg/day for 12 weeks) do not adversely affect thyroid function in oophorectomized women [62]. The results from a three-year, randomized, double-blind, placebo-controlled trial provided evidence that genistein supplementation is not associated with an increased risk of hypothyroidism after menopause [63]. Regular consumption of genistein aglycone (54 mg/day) did not influence fT4, fT3 and TSH concentrations and the levels of anti-TPO and anti-thyroglobulin antibodies were also unchanged. Furthermore, there were no significant differences in the expression of thyroid hormone receptors (THRα and THRβ) in peripheral blood mononuclear cells between the participants receiving genistein and the placebo [63]. Finally, the effects of consumption of low- (1.64 ± 0.19 mg isoflavones/day) or high-isoflavone (61.7 ± 7.4 mg isoflavones/day) soy protein isolate on T3, T4, TSH and TBG plasma concentrations did not differ in young men from those exerted by milk protein isolate given with the diet [64]. 

Lastly, it should also be pointed out that thyroid disrupting effects could be attributed solely to in vitro and animal studies [65,66]. According to the opinion of European Food Safety Authority (EFSA), there are no clinically relevant changes in thyroid function associated with consumption of soy protein, daidzein-rich isoflavones, genistein or red clover extract in post-menopausal women with normal thyroid function [67]. However, soy-based products might increase the daily dose of thyroid medication in hypothyroid patients, as they may interfere to some extent with the levothyroxine absorption. Drug regimens (taken on an empty stomach) exclude such risk [68].

The details of mentioned studies are summarized in Table 2 in order of appearance in the text.

## 4. Conclusions

The level and the type of dietary protein affect HPT axis activity. Protein restrictions are hazardous both in humans and animals, especially during pregnancy and/or lactation periods, since they exert long-term effects on the development, growth, metabolic and hormonal status of progeny. Although it was shown that isoflavones are able to inactivate TPO in vitro, the in vivo study excluded their direct relationship with hypothyroidism. Additional prerequisites like iodine deficiency or other goitrogenic dietary factors appear to be necessary for soy protein to alter thyroid axis activity.

## Figures and Tables

**Table 1:** Summary of selected studies investigating the effect of protein quantity on HPT axis activity.

Diets	Subjects	Duration	Main Results	Reference
Control: 18.3% protein, 67% carbohydrate, 5% fat; Protein-free diet: 0% protein, 83.1% carbohydrate, 5% fat; Protein/fat-free diet: protein: 0%, carbohydrate 99%, 0% fat;	CFY rats	12 days	Protein-free vs. control diet: lower hypothalamic TRH mRNA, pituitary TSHβ mRNA and plasma T3;	[14]
Control: 23% protein, 66% carbohydrate, 11% fat; Low protein (PR): 8% protein, 81% carbohydrate, 11% fat;	Wistar rats	during pregnancy and lactation	In adult offspring of PR mothers: lower muscle deiodinase (D) type 2, higher muscle D1, higher pituitary D2 activities without changes in thyroidal enzyme activities; Lower TSH response to in vitro TRH suggests a hyperthyroid status;	[20]
Control: 23% protein, 66% carbohydrate, 11% fat; Protein-restricted (PR): 8% protein, 81% carbohydrate, 11% fat; Energy-restricted (ER): a control diet in restricted quantities;	Wistar rats	during pregnancy and lactation	The PR group had significantly a higher thyroid 131I uptake, T3 and T4 serum concentrations compared to the controls;	[21]
Control: a standard laboratory diet, per 100 g: 22 g protein, 65 g carbohydrate, 5 g lipid and 5 celluloses; Protein-deprived: an isocaloric and protein-restricted diet with 8% protein;	Wistar rats	during lactation	Protein malnutrition of the dams during suckling resulted in hypothyroidism in the pups, lower radioiodine uptake followed by lower T3 and slightly higher TSH concentrations vs. controls;	[22]
Control: 23% protein, 66% carbohydrate, 11% fat; Protein-restricted (PR): 8% protein, 81% carbohydrate, 11% fat; Energy-restricted (ER): a control diet in restricted quantities;	Wistar rats	during lactation	The PR group: higher fT3 concentration, lower fT_4_ concentration, higher 24-h mammary gland and milk radioiodine (I^131^) uptake but lower 2- and 24-h thyroid I^131^uptake vs. controls; Protein deprivation during lactation did not affect thyroid or liver 5′-deiodinase activity;	[24]
Control: 23% protein, 66% carbohydrate, 11% fat; Protein-restricted (PR): 8% protein, 81% carbohydrate, 11% fat; Energy-restricted (ER): a control diet in restricted quantities;	Wistar rats	during lactation	In the PR and the ER group higher liver D1 activity compared to the control group; The PR group had a higher T3 and T4 concentrations and lower serum TSH level compared to controls;	[25]
Low (L): 70% High (H): 240% of National Research Council recommended crude protein (CP) requirements;	beef heifers	during gestation	Protein intake during the I and II trimesters of gestation has a gender-dependent effect on progeny thyroid hormone concentrations, which is associated postnatal growth pathway; L vs. H male progeny fT4 concentration at birth was higher; L vs. H for progeny independent of sex, greater fT3 relative to T3 at birth was recorded;	[26]
Control: modified the AIN 76 A with 19% protein, Low protein: isoenergetic with 8% protein;	Sprague–Dawley rats	during gestation and lactation	There were no differences in plasma T3 and T4 levels between control and low protein offspring in both fed and fasted states; Low protein (LP) offspring demonstrated hyperphagia mediated by alterations in leptin and ghrelin concentrations; LP offspring displayed higher resting and basal metabolic rates, higher core body temperature in both the fed and fasted states;	[27]
Control (C): standard laboratory diet with 23% protein. Protein-restricted (PR): isoenergetic with 8% protein;	Wistar rats	during gestation and lactation	Adult PR off spring demonstrated hyperthyroid state and lower thyroid JAK-2 expression, suggesting peripheral leptin resistance; In PR vs. C: T3 and T4 concentrations were higher and serum TSH levels were lower. Pituitary TSH content in PR off spring was no different from the control group; Adult PR offspring displayed lower hypothalamic STAT-3 expression vs. C but Ob-R but JAK-expression was similar; In the pituitary, PR offspring demonstrated higher Ob-R content vs. C, with unchanged JAK-2, STAT-3; In the thyroid gland, the PR vs. C presented lower JAK-2 content; but no change was observed in Ob-R, STAT-3 expression.	[28]
Eight dietary groups (total iodine intake µg/day): normal iodine control (NI, 4.65), 10-fold excess iodine (10HI, 46.5), 50-fold excess iodine (50HI 232.5), 100-fold excess iodine (100HI, 465), low-protein control (LC, 4.65); low-protein, 10-fold excess iodine (L10HI, 46.5); low-protein, 50-fold excess iodine (L50HI, 232.5) low-protein, 100-fold excess iodine (L100HI, 465) The protein/carbohydrate/fat ratio was 1:6:1 and 1:4:1 in the low-protein and normal protein groups, respectively;	Wistar rats	2, 4 and 6 months	At the end of 6 months, T4, fT4, T3 and fT3 in the low-protein excess iodine groups were lower than the groups with an equal amount of excess iodine alone; With increasing the iodine dosage relative weight of the thyroid gland increased; Goitres appeared in the high-dosage groups (100HI, L50HI and L100HI). Excess iodine produced damage to the ultrastructure of thyroid and apoptosis of follicular epithelial cells; in the low-protein excess iodine groups the degree of goitre formation was more pronounced;	[29]
Calorie restriction (CR): 23%protein, 49% carbohydrate and 28% fat; Sedentary subjects consuming a Western diet (WD): 17% protein, 52% carbohydrate and 31% fat; Endurance runners consuming a WD (EX): 15% protein, 53% carbohydrate and 32% fat;	age- and sex-matched healthy lean weight-stable volunteers	3–15 years (6 ± 3 years)	T3 concentration was lower in the CR vs. WD and EX whereas T4, fT4, reverse T3 and TSH concentrations were similar among groups;	[30]
Ad libitum (AL): a standard diet (100 g diet): 21.20 g protein, 70.60 g carbohydrate and 3.80 g; Caloric restriction with 20% deficit (CR20) (100 g diet): 26.40 g protein, 65.70 g carbohydrate and 3.50 g fat; Caloric restriction with 40% deficit (CR40) (100 g diet): 35.00 g protein, 57.50 g carbohydrate and 3.10 g fat;	Sprague-Dawley rats Two age groups: younger (17-week-old) and older rats (45-week-old)	9 weeks	fT3/T3, fT4/T4 ratios were affected by age and TSH, T4, T3 plasma levels were influenced by age; CR reduced fT3, T4 and fT4 concentrations in both age groups, with additional TSH and T3 decreases in 17-week-old rats; Regardless of age, cardiac myosin heavy chain â (BMHC) expression was positively correlated with cardiac deiodinase type 3 (D3) and negatively with food intake and thyroid hormone concentration;	[31]
Control: with a protein-energy: total energy value of 0.03 (23 g protein/kg/day); Six experimental groups: all with the same protein content (4.1 g/kg body weight/day) but reduced energy intake to either 90, 80, 70, 60 or 50% of the Control;	rats	2 weeks	T3 plasma concentration was elevated in animals with excess energy intake;	[32]
Control: with 180 g casein/kg diet; Low-protein: with 80, 45 and 0 g casein/kg fed ad libitum;	Sprague-Dawley rats		T3 increased in response to protein deficiency but fT3 decreased in response to low-protein diets	[33]
Normal protein (NP): 18% casein and 70% carbohydrate; Low protein (LP): 9% casein and 79% carbohydrate;	Wistar rats	4–8 weeks	In LP vs. NP T3 concentration was increased; in LP vs. NP fT4, T4 and TSH concentrations were reduced, suggesting that prolonged protein deficit may have an inhibitory effect on the HPT axis activity;	[34]
Control (C): standard diet with 18% protein; Protein-restricted (PR): with 6% protein; Energy-restricted (ER): control diet restricted to 60%;	Wistar rats	28 days	Serum T4 binding globulin (TBG) activity was increased and transthyretin (TTR) decreased in response to the dietary restrictions; Energy-deficient or protein-deficient diet caused up-regulation of TBG, down-regulation of TTR; PR vs. ER was related to increased T3 and fT3 concentrations;	[35]
Very-low-calorie (VLCD) high protein liquid diet—Optifast-70, 420 kcal/day: 70 g of casein and egg white protein, 30 g of carbohydrate, 2 g of fat;	Middle-aged, obese, euthyroid women	4–6 months	Resting metabolism rate (RMR, kcal/day), T3 and reverse T3 concentrations decreased significantly; T4 remained unchanged; After re-alimentation, all metabolic parameters increased significantly reaching pre-diet values, except for RMR and T3 levels;	[37]
Groups with different feeding frequency (FF) regime: 1 (FF1), 2 (FF2) or 4 (FF4) meals daily, each of two feeding level (FL): FL low and FL high; 1.5 and 2.5 × metabolizable energy requirements for maintenance (MEm), respectively;	Holstein-Friesian calves	28 days	T3 and T4 concentrations were modified by both FF and FL and were higher in FF4 at FLhigh vs. FLlow.	[39]

Highlighted lines provide data from human studies.

**Table 2:** Summary of selected studies investigating the effect of protein quality on HPT axis activity.

Diets	Subjects	Duration	Main Results	Reference
Semisynthetic isoenergetic based on: casein with amino acid supplementation (CAS+) (methionine + cysteine, threonine, tryptophan) or soy protein isolate without amino acid supplementation (SPI−) at the recommended protein level—100% (normal protein level, NP) and at a protein supply of 50% of NP (low protein level, LP)	Pigs	10 days of pre-period and 8 days of the main period	At NP, thyroid hormone (TH) concentrations were not affected by the dietary protein quality and after decrease of protein supply to 50% (LP) TH were dependent on the dietary protein quality; At LP T4 concentration increase was recorded in both protein quality groups, T3 and fT3 concentrations were higher in CAS+ vs. SPI-;	[44]
Semi purified isonitrogenous and nearly isoenergetic diets with 10% protein: casein supplemented with methionine (casein rats), soy protein isolate without supplements (soy protein rats), soy protein supplemented with methionine (supplemented soy protein rats)	Fischer rats	30 days	T4 level was lower in soy protein rats vs. casein rats; The fT4 levels were decreased in soy protein- and supplemented soy protein rats vs. casein rats; There were no differences in T3 and fT3 concentrations between the dietary groups;	[45]
Experiment 1: casein (222.2 g casein/kg diet) with isoflavone supplementation (0, 5, 50, 250 and 1250 mg/kg diet), alcohol-washed soy protein isolate (SPI, 222.2 g soy protein/kg diet) with isoflavone supplementation (0, 5, 50, 250 and 1250 mg/kg diet) Experiment 2: casein (307 g/kg diet) casein (307 g/kg diet) plus isoflavones supplementation (50, 100, 200, 400 mg/kg diet)	Sprague-Dawley rats	Exp. 1: 70, 190 and 310 days Exp. 2: 120 days	TRα1, TRα2 and TRβ2 contents in the liver were not affected by SPI; The level of the hepatic TRâ1 protein was increased by dietary SPI in both sexes vs. casein; TRâ1 was not affected by added isoflavones but isoflavones in high doses (250 and 1250 mg/kg diet) reduced the hepatic TRβ1 protein content in F1 male rats on d 28; SPI had no effect on total T3 and T4 levels; higher doses of isoflavones caused T4 increase in female rats;	[46]
Protein concentration 25 g/100 g diet and three sources: isolated soy protein (ISP), soy protein concentrate (SPC), casein	Golden Syrian hamsters	35 days	T4 and fT4 concentrations were higher in ISP vs. casein; T3 concentration was higher in casein-fed vs. SPC-fed hamsters; ISP and SPC were both effective in reduction blood cholesterol concentrations;	[47]
Control: standard NIH 31 diet, Soy—free diet fortified with genistein aglycone at various levels (0, 5, 100, or 500 mg/g diet)	Sprague-Dawley rats	140 days	TPO activity was reduced by up to 80% in a dose-dependent manner; TPO activity was approximately 50% lower in Controls vs. soy-free group and this reduction was corresponding to serum levels of isoflavone; T3, T4, TSH concentrations, thyroid weights and histopathology exhibited no differences between groups;	[52]
Control: vehicle, i.e., a dimethyl sulphoxide (DMSO):water mixture (4:6 *v*/*v*), Genistein dissolved in the vehicle—1 mg/kg BW Genistein dissolved in the vehicle—5 mg/kg BW; The vehicle and genistein solutions were administrated intragastrically (0.5 mL/100 g BW/once a day)	Wistar rats	7 days	T4, fT4 and T3, fT3 concentrations were not affected by genistein;	[54]
Soy (34.3% protein), Soy-free (34.2% protein, Poultry by-product—(the main source of protein)	Cats	3 months	At soy diet vs. soy-free diet: T4 and fT4 concentrations were higher but unchanged T3 concentrations;	[55]
Habitual diets supplemented with 3 soy protein powders (348 kcal: 63 g protein, 21 g carbohydrate and 1.9 of fat) with a different isoflavones concentration: Control: 0.11 ± 0.01 mg/kg BW/day, Low isoflavone (low-iso): 1.00 ± 0.01 mg/kg BW/day; High isoflavone (high-iso): 2.00 ± 0.02 mg/kg BW/day]	Healthy postmenopausal women	93 days	No significant effects of isoflavone consumption were observed for TSH or thyroid hormones; Thyroid binding globulin (TBG) was significantly decreased by the high-iso vs. low-iso and control diet;	[56]
A soy-free diet with increased iodine content (1.1 mg iodine/kg diet): Control: vehicle solution 10 mg/kg BW of genistein (G), 10 mg/kg BW of daidzein (D); all substances were injected subcutaneously;	Orchidectomized Wistar rats	3 weeks	TSH level increased and T3 and T4 concentrations decreased in both isoflavone-treated groups vs. controls; Both G and D stimulate microfollicular changes in the thyroid tissue and reduce the level of thyroid hormones; In both isoflavone-treated groups vs. controls thyroid tissue had increased: volume of thyroglobulin-immunopositive epithelium, epithelial height and index of activation rate but reduced the volume of luminal colloid;	[57]
Diets with different protein (19%) sources: Casein-lactalbumin (CL), Soy protein with isoflavones (SOY);	Cynomolgus monkeys	68 months	In pre-ovariectomized monkeys T3 concentration higher in SOY vs. CL; In post- ovariectomized SOY prevents a decline in serum T4 level; Both soy protein and isoflavone consumption may preserve thyroid function menopause;	[58]
Group I: 3.5 kg of 100% red clover silage, Group II (Control): hay;	Swedish Finewool Landrace ewes	14 days	T3 and fT3 was higher in Group I vs. II; Thyroid follicles were larger and Era immunoreactivity was stronger in thyroid glands in ewes fed red clover silage vs. control;	[59]
Habitual diets supplemented with: Maltodextrin (Placebo), or an isoflavone supplement with 50 mg soy isoflavones;	Healthy postmenopausal women	90 and 180 days	There were no significant differences in TSH, T4 and T3 concentrations between isoflavone supplement and placebo groups;	[60]
40 g protein as part of a National Cholesterol Education Program Step I diet was provided as: ISP containing 56 mg isoflavones (ISP56), ISP containing 90 mg isoflavones (ISP90), casein (0 mg total isoflavones/g protein);	Hypercholesterolemic postmenopausal women	6 months	T4 and fT4 concentrations were higher in the ISP56 vs. control; TSH and T3 concentrations were higher in the ISP90 group vs. control;	[61]
A habitual diet supplemented with: tablet with 75 mg of soy isoflavones and 200 mg elemental calcium, 100 mg elemental phosphorus and 100 IU of vitamin D3; Placebo tablet matching in colour, size, shape and taste and containing identical quantity of calcium, phosphorus and vitamin D3;	Oophorectomized women	12 weeks	Modest reduction in fT3 concentration in the isoflavone group was recorded; The mean change in other thyroid parameters was not significantly different between the two groups;	[62]
Isocaloric standardized, fat-reduced diet supplemented with: Tablet with 54 mg of genistein aglycone plus calcium and vitamin D3 at therapeutic doses, Placebo tablet plus calcium and vitamin D3 at therapeutic doses;	Postmenopausal women	3 years	Genistein administration did not influence thyroid hormones or autoantibodies concentrations; There were no differences in THRα, THRβ, RARα, RARγ, or RXRα mRNA expression between groups;	[63]
Habitual diets supplemented with: Milk protein isolate (MPI), Low-isoflavone soy protein isolate (low-iso SPI; 1.64 ± 0.19 mg iso/day), High-isoflavone SPI (high-iso SPI; 61.7 ± 7.4 mg iso/day);	Healthy men	57 days	There were no differences between low-iso and high-iso SPIs in T3, fT3, T4, fT4, TSH, or TBG concentrations when compared with the MPI;	[64]

Highlighted lines provide data from human studies.
